# Gallium phosphide optical metasurfaces for visible light applications

**DOI:** 10.1038/s41598-020-77753-0

**Published:** 2020-11-26

**Authors:** Mauro Melli, Melanie West, Steven Hickman, Scott Dhuey, Dianmin Lin, Mohammadreza Khorasaninejad, Chieh Chang, Sunny Jolly, Huy Tae, Evgeni Poliakov, Pierre St. Hilaire, Stefano Cabrini, Christophe Peroz, Michael Klug

**Affiliations:** 1 Magic Leap Inc., Plantation, FL 33322 USA; 2 The Molecular Foundry, Lawrence Berkeley National Laboratory, Berkeley, CA 94720 USA

**Keywords:** Optics and photonics, Metamaterials, Metamaterials

## Abstract

There are few materials that are broadly used for fabricating optical metasurfaces for visible light applications. Gallium phosphide (GaP) is a material that, due to its optical properties, has the potential to become a primary choice but due to the difficulties in fabrication, GaP thin films deposited on transparent substrates have never been exploited. In this article we report the design, fabrication, and characterization of three different amorphous GaP metasurfaces obtained through sputtering. Although the material properties can be further optimized, our results show the potential of this material for visible applications making it a viable alternative in the material selection for optical metasurfaces.

## Introduction

Within the last decade, optical metasurfaces in the visible range have been raising more and more expectations as potential substitutes for refractive optics in many applications, due to their benefit in terms of size, weight, and cost^1–4^. Metasurfaces, arrays of nanoantennas or optical resonators, are dielectric or plasmonic structures that allow control of the amplitude, phase, and polarization of light by locally modulating its wavefront. The scattering response of individual antennae depends on their geometry and materials. Great effort has been invested in developing new geometries and designs^1–7^ but the choice of material is still limited. Pioneering work was done on subwavelength structures in titanium oxide^8^, and the field became very popular a few years later after the publication of gold-based plasmonic metasurfaces^9^. However, the high loss of plasmonic structures and resulting low efficiencies have limited the expansion of optical metasurfaces to imaging applications. As promising alternatives, semiconductors, and dielectric materials, with their relative low absorptivity, have been used to demonstrate metasurfaces working within the near-infrared and visible ranges. Lin et al.^10^ published the first result of silicon-based metasurfaces. Khorasaninejad et al*.*^11^ demonstrate high efficiency metalenses across the visible range and later a large variety of optical components, using a novel fabrication process to create high aspect ratio titanium oxide metasurfaces. Although other materials^12–14^ have been proposed and reported as the base material for making metasurfaces, none of them have allowed to achieve similar optical performances as the ones reached with silicon and titanium oxide, especially in the visible range. However, both materials have their own downsides. Silicon has a large index of refraction, but exhibits significant absorption, which limits its optical performance. On the other hand, titanium dioxide is transparent, but its index of refraction is relatively low, which requires nanostructures with a high aspect ratio to tune the phase shift over the full 0–2π range. This makes the fabrication of these structures more challenging.

Gallium phosphide (GaP) is a semiconductor material that offers great potential for developing metasurface-based devices in the visible domain. As a single crystal, it has an indirect bandgap of around 2.24 eV^15^. As a result, GaP is transparent in a large region of the visible spectrum (wavelength λ > 0.54 µm). The index of refraction is higher than 3.1 in the full range of visible domain (n = 3.6 at 500 nm wavelength)^16^. This value is not as high as the one of silicon (n = 4.3) but it is significantly higher than other transparent material such as titanium oxide (n = 2.4–2.7), gallium nitride (n = 2.4), and hafnium oxide (n = 1.9). All these properties present GaP as an extremely attractive alternative to both silicon and titanium dioxide.

GaP is not a novel material in optics and it has been used in Light-Emitting Diodes (LED) manufacturing for decades^17^ but its use for nanophotonics devices is very limited. GaP thin films have been used for demonstrating photonic crystal nanocavities with high optical performances (quality factors as high as 1700)^18^, and, optical nanoantennas have been fabricated into GaP substrates for surface-enhanced harmonic generation and fluorescence with low loss in the visible range^19,20^. The potential of gallium phosphide in designing metalens has been proposed and simulation results have been reported before^21^ but, to the best of our knowledge, GaP has not been experimentally used as a material for metasurface-based optical elements for visible light applications, due to the challenges both of growing or depositing thin films on transparent substrates, and nanopatterning GaP films. Indeed, it is commonly accepted that only high-quality epitaxially-grown films can be used for making a nanophotonic devices.

In this paper, we report an alternative strategy to fabricate optical metasurfaces based on GaP thin films. We have chosen three metasurface designs not only with different functions but also with different geometrical properties, such as pattern density and pattern resolution, to cover distinctive fabrication regimes. Two transmission gratings based on different design concepts and a lens, have been developed to demonstrate the potential of this approach for applications in the visible range.

## Metasurface design principle

Optical elements manipulate the wavefront of the interacting light and, in many cases, can be described by a space-dependent phase shift function. For example, blazed gratings are characterized by a linear phase profile. Wrapping the phase values in the 0–2π range, the phase profile variation follows the following equation:

$${\varphi }_{d}\left(x\right)={\left.\left(\frac{2\pi }{d}\right)*x\right|}_{mod2\pi }$$

where d is the period of the grating. The behavior of a simple lens is given:

$$\varphi \left(x,y\right)={\left.\frac{2\pi }{\lambda }\left(f-\sqrt{{x}^{2}+{y}^{2}+{f}^{2}}\right)\right|}_{mod2\pi }$$

where λ is the wavelength and f is the focal length.

Metasurfaces, the subwavelength arrangement of arrays of nanoantennas or optical resonators, can be used to create optical elements with customized phase shift response. The continuous phase shift functions are discretized in unit cells which contain individual resonators. High refractive index semiconductor nanobeams can produce strong electrical and/or magnetic resonances in the visible range^22^. The resonance features depend on the geometry of the nanobeams, specifically the height, width, and length. This property can be used to design metasurface gratings. In a possible configuration, infinitely long nanobeams are arranged with subwavelength spacing. The height is given by the film thickness and thus the width is the only geometrical parameter that can be modulated to tune the resonance frequency. Because different resonances induce different phase delays it is possible to modulate the phase front by arranging nano beams with different width. A second approach, known as Pancharatnam-Berry Optical Elements (PBOE), is based on the subwavelength arrangement of polarization-dependent elements that add a space-dependent geometric phase which, as in the previous approach, allows manipulation of the wavefront^23^.

**Figure 1:**
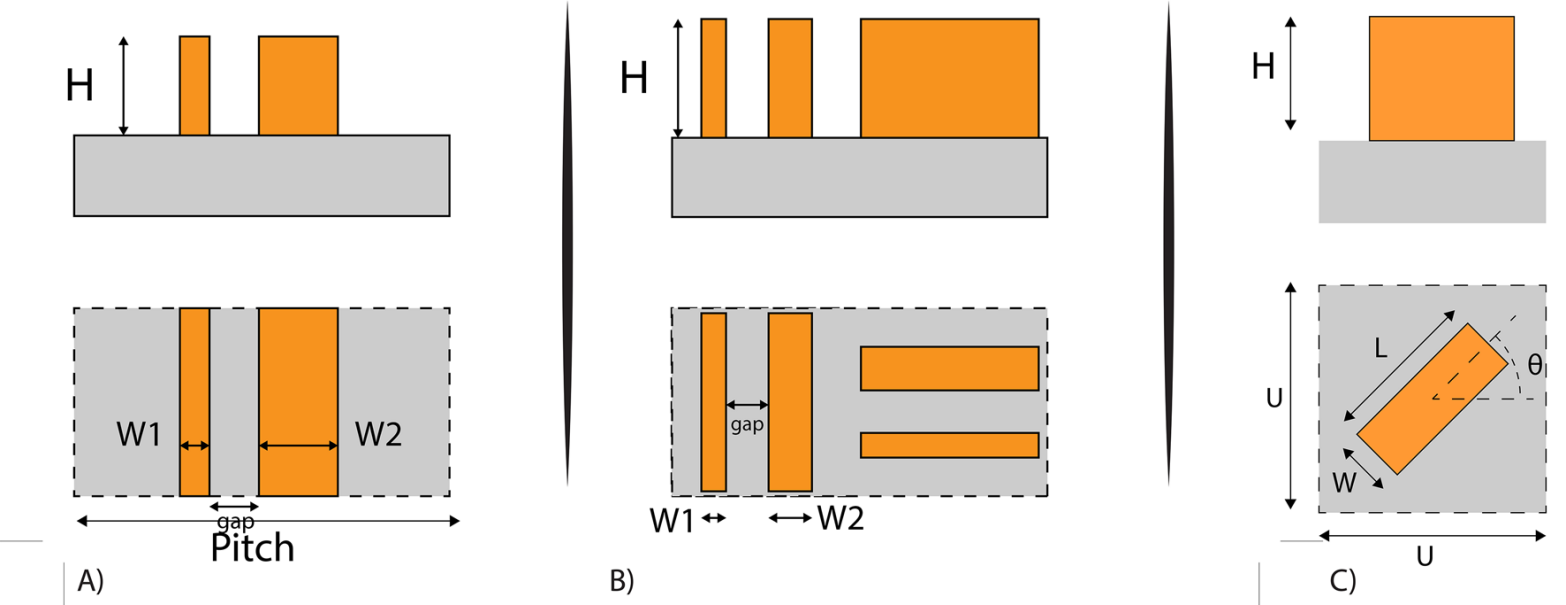
Schematics of metasurface design. (**A**) Asymmetric grating (blaze grating phase profile) (**B**) PBOE grating (blaze grating phase profile) (**C**) Metalens unit cell.

The first design (Fig. 1A) is similar to the one we previously reported using silicon infinitely long nanobeams^7^ and is used to reproduce the phase profile of a transmissive blazed grating. The pitch of the grating (p = 380 nm) is chosen to maximize the diffraction angle at wavelength of 520 nm. Because of the high spatial frequency, it is hard to have more than three-phase levels within one grating pitch.

The second design of transmissive blazed grating is a Pancharatnam-Berry Optical Elements (PBOE), where a geometric phase modulation is implemented by rotating the unit cells with respect to each other. The unit cell consists of two different widths of nanobeams and has a size of 190 nm × 190 nm. As in the previous design, the pitch of the grating is relatively short (p = 380 nm), and only two orientations can be fitted into one grating period (Fig. 1B). In the case of a symmetric structure unit cell, the metasurface will be mirror symmetric, and therefore will equally diffract light to both + 1 and − 1 orders. Conversely, introducing non-symmetric nanobeam widths in the unit cell allows for higher efficiency in the desired diffraction order.

The third design is a metalens (Fig. 1C). The building blocks of the design are GaP single nanobeams. Using the geometric phase concept, the phase at each coordinate is realized via rotation of the nanobeam by an angle θ(x,y). For a right-handed circularly polarized light, these rotations yield a phase shift having twice of the rotation angle:

$$\mathrm{\varphi }\left(\mathrm{x},\mathrm{y}\right)=2\uptheta \left(\mathrm{x},\mathrm{y}\right).$$

This phase shift is associated with a polarization conversion to left-handed circularly polarized light. The maximization of the focus efficiency is obtained by maximizing the polarization conversion efficiency of each nanobeam which are used as a half-waveplate.

Even though the fabrication requirements are very different as stated in the introduction, the fabrication process flow does not depend on the specific design and it is described in the next section.

## Metasurface fabrication

### Film deposition

Growth of single crystal GaP films by either molecular beam epitaxy (MBE)^24^ or metal–organic chemical vapor deposition (MOCVD)^25^ can be done only on a very limited number of substrates (such as silicon). It cannot be performed on most transparent substrates for visible light applications, such as glass or sapphire wafers, because of mismatch of the lattice parameters. Recently, an approach based on the epitaxial lift-off technique has been proposed to overcome this limitation^26^. Single crystal GaP thin films are first growth on silicon wafers. Subsequently the silicon wafers are bonded to a glass wafer using SU8. The silicon growth substrates are then dissolved with a xenon difluoride vapor etch. Though this process produces films with excellent optical properties, it is complex, and it has not yet been demonstrated on an area larger than 1 in^2^. A cost-effective and scalable approach to deposition of GaP thin films is by RF sputtering. A few works have reported on the feasibility of depositing amorphous GaP films for optical applications^27,28^. Optimization of the deposition conditions allows one for obtaining high index, low loss amorphous GaP thin films. In the current work, we have extended these initial results to deposit GaP thin films on glass and sapphire wafers with optical and morphological properties suitable with the development of metasurfaces.

**Figure 2:**
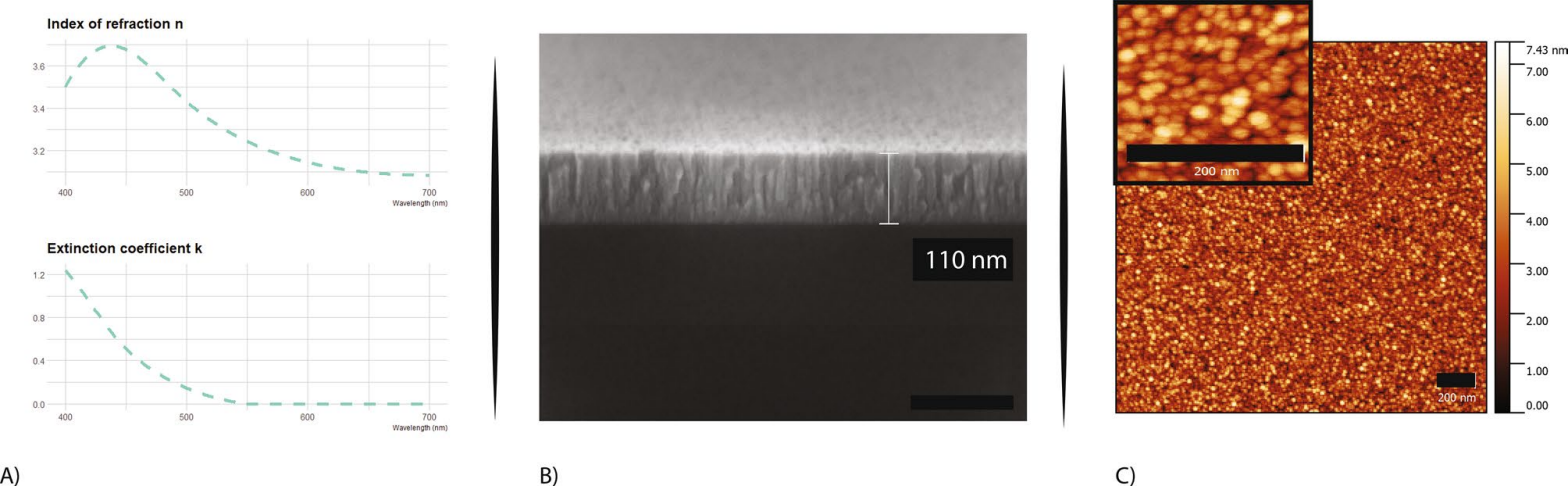
GaP film characterization. (**A**) Ellipsometry Measurements of the index of refraction and extinction coefficient in function of the wavelength. (**B**) SEM cross-section of a GaP film deposited on silicon. (**C**) AFM scans. Scale bar 200 nm.

The GaP films were deposited by a load-locked Kurt J. Lesker Lab18 RF sputtering system. The sputter target consists of a 3-inch GaP wafer (99.9% pure) elastomer bonded to a copper backplane. Depositions were performed with RF power at 100 W and chamber pressure of 7.5 mTorr, resulting in a deposition rate of around 0.5 A/s. Silica and sapphire wafers were used as substrates and were pre-cleaned by RCA process^29^. Different deposition temperatures were tested ranging from room temperature up to 550 °C. It was previously reported that post-deposition annealing can restore the correct stoichiometry and induce a partial re-crystallization of the material, resulting in improved optical properties of the film^28^. For this reason, a post-deposition rapid thermal annealing (RTA) step was performed at higher temperatures ranging from 600 to 1000 °C. The temperatures of the deposition and of the RTA strongly influence the refractive index, n, and the extinction coefficient, k, of the deposited films. Increasing the temperature during the deposition results in an increase of the refractive index and a reduction of the extinction coefficient. The metasurfaces presented here were made by films deposited at temperatures of 450 °C and 550 °C. The chosen temperature for the RTA step was 750 °C for 5 min. This last step is possible only for substrates which can handle a such high temperature. Examples of a Scanning Electron Microscope (SEM) cross-section and Atomic Force Microscopy (AFM) scans are shown in Fig. 2B,C. The measurement of the optical properties (Fig. 2A) was performed by ellipsometry, with n ranges from 3.8 to 3 in the wavelength range between 450 to 800 nm, and k is 0 for wavelengths greater than 560 nm. The final GaP films are smooth with roughness below 1 nm (RMS value).

## Metasurface fabrication

**Figure 3:**
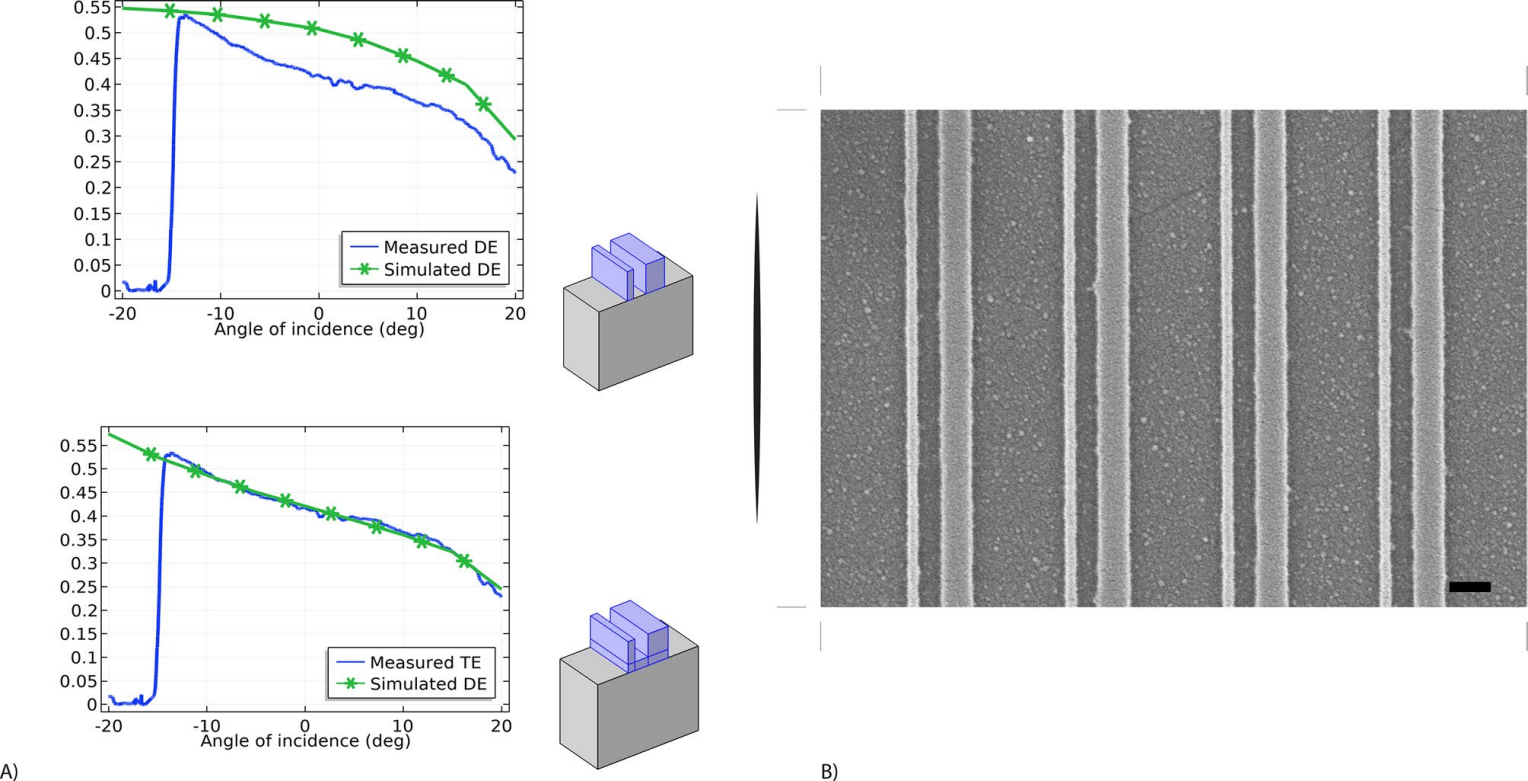
Asymmetric grating. (**A**) Experimental and Simulated Diffraction Efficiency of the first diffracted order T1 (TM mode) at the wavelength of 520 nm. The cutoff at − 15 deg is due to the index mismatch between the sapphire substrate (n = 1.8) and the index matching oil used for that measurement (n = 1.56). See Supporting Information, for details. Both the simulation with and without a residual layer in the gap are shown. (**B**) SEM top view. The sample was metalized for SEM imaging. Scale bar 200 nm.

**Figure 4:**
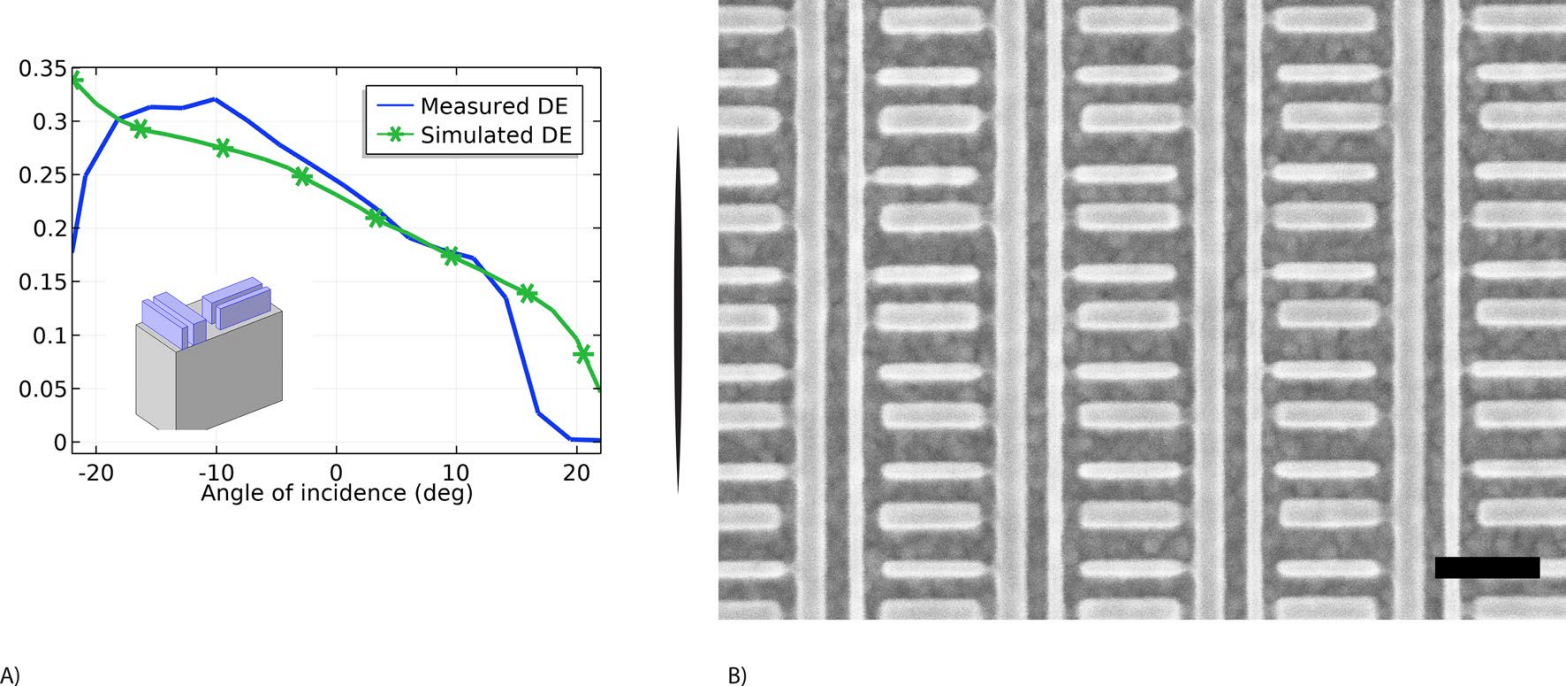
PBOE grating. (**A**) Experimental and Simulated Diffraction Efficiency of the first diffracted order T1 (TM mode) at the wavelength of 520 nm. (**B**) SEM top view. The sample was metalized for SEM imaging. Scale bar 200 nm.

**Figure 5:**
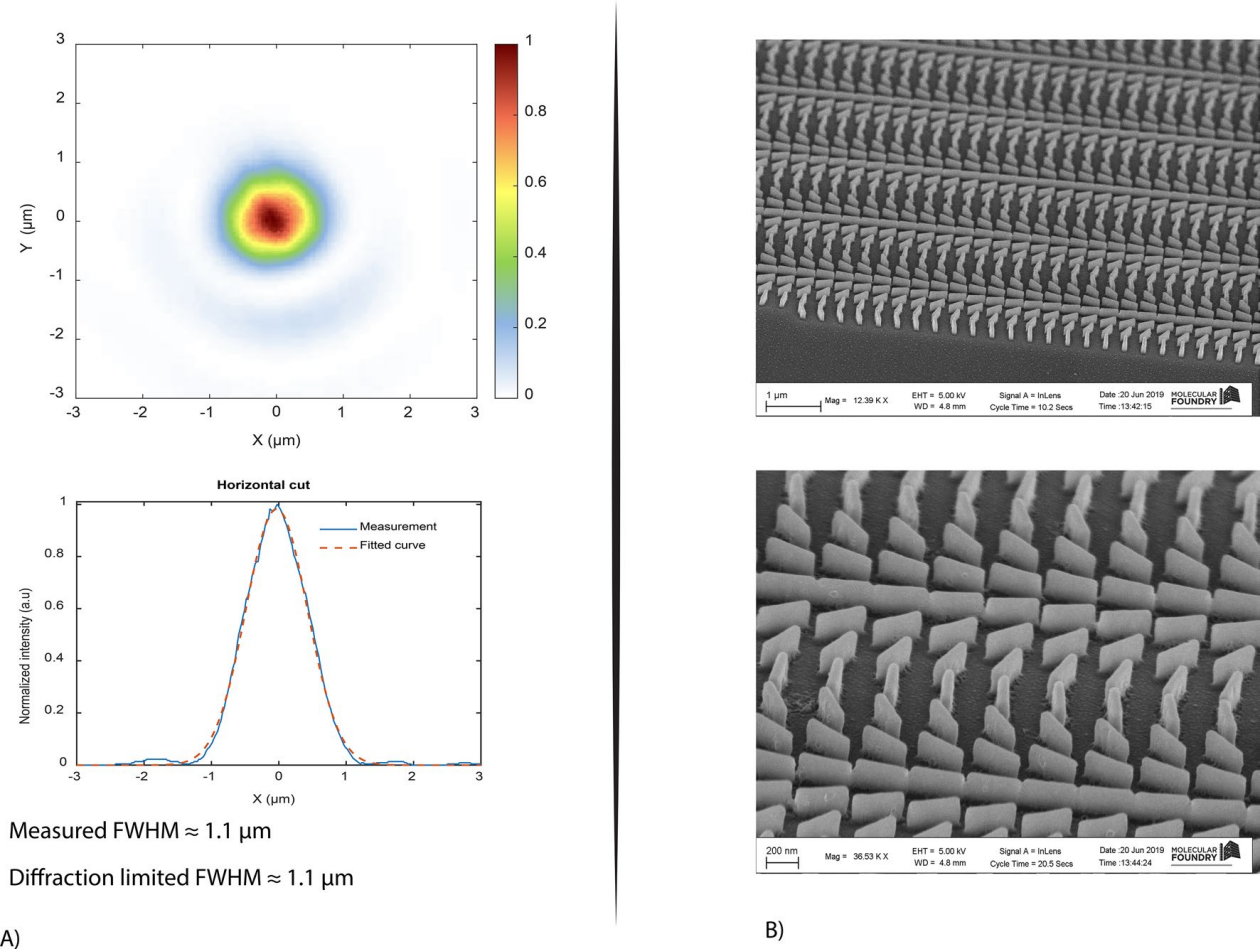
Metalens: (**A**) Measured spot size in the focal point at the wavelength of 450 nm. (**B**) SEM images of the metalens. The sample was metalized for SEM imaging.

After the GaP film deposition, the wafers were spin-coated with a layer of e-beam resist hydrogen silsesquioxane (HSQ). A conductive layer of aquaSAVE (from Mitsubishi Rayon) was applied to reduce electrostatic effects associated with the insulating wafers to avoid electric charge accumulation during the exposure. Patterning was performed by electron beam lithography (Vistec VB300) at an accelerating voltage of 100 kV and a beam current of 2 pA. The total exposed area for all the metasurfaces was between 0.25 and 1 mm^2^. After the exposure, the aquaSAVE layer was removed by rinsing the sample in DI water. Salty development in an aqueous mixture of 1 wt% NaOH and 4% wt NaCl for 4 min was used to reach a high contrast and resolution down to 10 nm^30^. The patterns were transferred into the GaP film using Inductively Coupled Plasma—Reactive Ion Etching (ICP-RIE) using a process previously reported^31^. The etching rate of GaP is around 25 nm/min. Because of the low influence of the remaining HSQ mask layer (thickness around 5–10 nm) on the optical properties, the remaining HSQ was not removed to avoid any damage to the structures or substrate. SEM images of the metasurfaces are displayed in Figs. 3B, 4B, 5B. The current process could be scaled to large area by using other lithography techniques such as immersion lithography or nanoimprint lithography^32^.

## Results

Finite element simulations were used to calculate and maximize the diffraction efficiency of the metasurfaces. We used the software COMSOL 5.4 to simulate the response of the unit cell. Depending on the symmetry of the problem, two-dimensional or three-dimensional simulations were carried out. For the first design of the asymmetric gratings, the line widths of nanobeams and gaps between nanobeams were optimized to provide the highest constant efficiency across a large range of incidence angles ([− 20;20] degrees). For the TM polarization, simulations showed that it is possible to achieve diffraction efficiency values ranging from 40 to 50% for the first diffracted order T1 over the targeted angular range (Fig. 3).

According to this design the values of the nanobeams widths are 25 nm and 75 nm and an edge-to-edge spacing of 55 nm for a 110 nm thick film. A 1 mm × 1 mm grating was fabricated and measured in a custom-built goniometer (similar to the previously described in ref.^7^, see SI). We have observed a discrepancy between the simulation of perfect metasurfaces and their measured diffraction efficiency, suggesting that the GaP material in the gap was not fully cleared during the etching because of different etching rates in the two gaps from micro loading effects. Longer etching will damage the substrate surface in the larger gap and would require a thicker resist making the high-resolution patterning more challenging. Adding a 30 nm residual layer in the gap between the two beams resulted in a much better reproduction of the experimental data (Fig. 3A). Although the diffraction efficiency is found to be slightly lower than the one for the “ideal design”, GaP asymmetric gratings show a significant improvement compared to the same design in silicon that we previously reported^7^ where the transmission mode showed relatively flat diffraction in the 30% range. We attribute the improvement to the lower extinction coefficient of the GaP film in the visible range.

The second design of a blazed grating (PBOE) is based on patterns with a higher resolution, down to 20 nm in a very dense pattern (Fig. 4B) making both the EBL patterning and the etching more challenging. This design allows for creating polarization-sensitive gratings with a high polarization extinction ratio (the ratio between the diffraction efficiency in two orthogonal polarizations) ranging from 60:1 to 15:1 (see Figure S3) and at the same time maintaining a high diffraction efficiency, up to 30% for TM mode (Fig. 4A).

The last design, a metalens, was designed and fabricated at the working wavelength of 450 nm (see Figure S4). The diameter of the lens is 1 mm and its focal length is 2.4 mm (NA = 0.2). Figure 5B shows the SEM image of a fabricated lens. For this specific design, the unit cell period is 350 nm and the dimension of the nanobeams are 315 nm × 45 nm × 135 nm (length × width × height). The measurement setup was identical to the one described in ref. ^1^. Figure 5A shows a highly symmetric focal spot that is obtained for the metalens at its designed wavelength. The lens works at the diffraction limit showing a spot size (FWHM) of 1.1 micron $\left(\frac{\lambda }{2NA}\right)$. The lens works close to the diffraction limit at other wavelength (See Supporting Information, Figures S5 and S6). The measured diffraction efficiency at 450 nm is 14.5% when the lens is illuminated with the right circularly polarized light and 1.2% in the case of left circularly polarized light. The GaP film used for this design did not go through the RTA step because the glass substrate could not withstand it. The resulting film has a higher k than the ones in the previous examples. For this reason, the measured efficiency is lower than the expected value (about 40%, see Figure S4).

In conclusion, we have reported here for the first time the feasibility of fabricating metasurfaces based on GaP thin films suitable for optical application in the visible range. Although future development and optimization of the material deposition method are required to achieve higher performance, we believe that GaP has the potential to become the primary photonic material choice in designing the next generation of metasurfaces in the visible range.

## Supplementary information

Supplementary Information.
