# Supplementary material for: Gallium phosphide optical metasurfaces for visible light applications
Source: Sci Rep. 2020 Nov 26;10:20694. doi: 10.1038/s41598-020-77753-0 (PMC7691993; doi:10.1038/s41598-020-77753-0)
Supplement: Supplementary file 1 — Supplementary Information. [file 41598_2020_77753_MOESM1_ESM.docx]

**Gallium phosphide optical metasurfaces for visible light applications – Supporting Information**

Mauro Melli^1,*^, Melanie West^1^, Steven Hickman^1^, Scott Dhuey^2^, Dianmin Lin^1^, Mohammadreza Khorasaninejad^1^, Chieh Chang^1^, Sunny Jolly^1^, Huy Tae^1^, Evgeni Poliakov^1^, Pierre St. Hilaire^1^, Stefano Cabrini^2^, Christophe Peroz^1^, and Michael Klug^1^.

^1^Magic Leap Inc., Plantation, FL, 33322, USA.
^2^The Molecular Foundry, Lawrence Berkeley National Laboratory, Berkeley, CA, 94720, USA.
^*^Correspondence and requests for materials should be addressed to M.M. (email: [mmelli@magicleap.com](mailto:mmelli@magicleap.com))

**Refractive index of Si, TiO2, and GaP (bulk values)**

Figure S1: Index of refraction and extinction plot of silicon^1^, titanium oxide^2^, and gallium phosphide^3^.

**Diffraction efficiency simulations**

We used the software COMSOL 5.4 with RF module. To simulate the grating, it is enough to simulate a single cell and applied periodic conditions (Floquet). The RF module has a built-in function to calculate the power transmission in all the allowed diffraction orders. The optimization is done by spanning the geometrical parameters on large range. Similarly, the efficiency of the lens is maximized by maximizing the polarization conversion efficiency of the single nanobeams.

**Diffraction efficiency experimental setup**

To obtain the diffraction efficiencies experimentally, we used a laser scanning approach as shown in Figure S2 where the laser beam was initially aligned at the center of the sample (focus spot of 120 um obtained through a custom designed focusing optics). The light is scanned by a commercially available galvanometric laser scanning assembly. The diffracted light then passes through a high-index truncated half-sphere (SF6 material) and collected by 5” integrated sphere (Labsphere) with a Thorlabs photodetector PMD100. A particular state of polarization was achieved by half- and quarter wave achromatic plates (Edmund Optics) installed into the scanning optics train. The apparatus was initially carefully calibrated to take into account the absorption of the optics, as well as the Fresnel reflections between the sample substrate and the half-sphere surface. The Fresnel losses between the GaP substrate and the truncated glass sphere were minimized by either a n = 1.56 (Figure 3A ) or a n=1.68 (Figure 4A) index matching fluid.

**
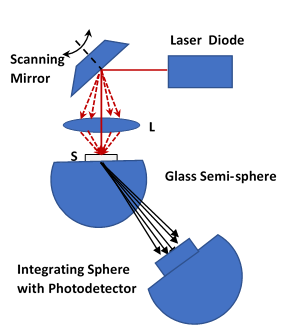
**

Figure S2: Schematics of a scanning goniometer used for characterization GaP samples: The light from a laser diode was focused and redirected by a scanning mirror to perform multiple incident angle of sampling. The diffracted light was then collected by a 2” input port integrating sphere with a photodetector. The entire set-up was controlled by a Labview program running Windows7.

**Diffraction efficiency TE vs TM of the PBOE grating**

Figure S3: Measured diffraction efficiency of the PBOE gratings at 520 nm.

**Metalens optimization.**

Figure S4: Conversion efficiency as a function of nanobeam width and length with incremental step of 10 at wavelength of 450 nm. Height is 135 nm. For our design we select L= 315 nm and W= 45 nm.

**Gap**

**Sapphire**

**H**

**L**

**W**

**Focal Spots at 520 nm and 635nm.**

Measured FWHM=~1.19 mm

Diffraction limited FWHM=1.09 mm

Figure S5: Measured spot size in the focal point at the wavelength of 520 nm.

Figure S6: Measured spot size in the focal point at the wavelength of 635 nm

Measured FWHM=~1.41 mm

Diffraction limited FWHM= ~0.95 mm

**References**

1. C. Schinke, P. C. Peest, J. Schmidt, R. Brendel, K. Bothe, M. R. Vogt, I. Kröger, S. Winter, A. Schirmacher, S. Lim, H. T. Nguyen, D. MacDonald. Uncertainty analysis for the coefficient of band-to-band absorption of crystalline silicon. AIP Advances **5**, 67168 (2015)
2. G.E. Jellison Jr. Optical functions of GaAs, GaP, and Ge determined by two-channel polarization modulation ellipsometry, Opt. Mat. **1**, 151-160 (1992)
3. J. R. Devore. Refractive indices of rutile and sphalerite, J. Opt. Soc. Am. **41**, 416-419 (1951)
